# Influence on study outcomes of an inpatient study by the behavior of the study staff (PINgPOng): study protocol for a randomized clinical trial

**DOI:** 10.1186/s13063-022-06436-0

**Published:** 2022-06-13

**Authors:** Martin Coenen, Ulrike Bingel, Matthias Zunhammer, Maria Soledad Berdaguer, Christine Fuhrmann, Rolf Fimmers, Jens Rengelshausen, Gunther Hartmann, Manfred Schedlowski, Christoph Coch

**Affiliations:** 1 Clinical Study Core Unit, Study Center Bonn (SZB), University Hospital Bonn, Sigmund-Freud-Str. 25, 53127 Bonn, Germany; 2 Institute of Clinical Chemistry and Clinical Pharmacology, University Hospital Bonn, Sigmund-Freud-Str. 25, 53127 Bonn, Germany; 3 Department of Neurology, University Hospital Essen, Hufelandstraße 55, 45147 Essen, Germany; 4 Institute of Medical Biometrics, Informatics and Epidemiology, University Hospital Bonn, Sigmund-Freud-Str. 25, 53127 Bonn, Germany; 5 Clinical Science, Grünenthal GmbH, Zieglerstr. 6, 52078 Aachen, Germany; 6 Institute of Medical Psychology and Behavioral Immunobiology, University Hospital Essen, Hufelandstraße 55, 45147 Essen, Germany; 7 Department of Clinical Neuroscience, Osher Center for Integrative Medicine, Karolinska Institutet, 171 77 Stockholm, Sweden; 8 nextevidence GmbH, Balanstraße 71a, 81541 Munich, Germany

**Keywords:** Placebo effect, Placebo response, Analgesic therapy, Study team

## Abstract

**Background:**

The placebo effect as the symptom improvement following inert treatments is a fixed component of RCTs to differentiate between specific effects of the tested pharmacological substance from other unspecific effects. The PINgPOng study was set up to analyze the influence of a study team trained to either minimize the placebo response and optimize drug-placebo differences or to maximize the placebo response to increase drug efficacy by unspecific factors on the study results of a RCT in a classical early clinical trial setting.

**Methods/design:**

PINgPOng is a single-center, prospective, randomized, double-blind, placebo-controlled study in a 3-group, 2-sequence, 2-period cross-over design. The study is conducted according to the principles of ICH-GCP and the Declaration of Helsinki on the Phase I-Unit of the University Hospital Bonn. The primary endpoint is the pain intensity in the cold pressor test before and after the administration of 15 mg oxycodone or placebo. The pain intensity is compared between three study conditions: 32 healthy volunteers in each study arm will be treated either by an untrained study team (arm A), by a study team trained to maximize (arm B), or to minimize placebo responses (arm C). Neuroendocrine factors (alpha-amylase activity, salivary cortisol), characteristic traits (anxiety, depression, stress), and somatic reactions are analyzed as covariates of the pain perception.

**Discussion:**

The PINgPOng study will allow to answer the question whether and to what extent the behavior of a trained study team (neutral vs. maximize vs. minimize placebo responses) will differentially affect placebo responses in a setting of a highly standardized early clinical trial. The results will help to control the placebo effects by education of the clinical study team and to avoid unnecessary high placebo effects in clinical development.

**Trial registration:**

German Clinical Trials Register DRKS00013586. Registered on December 22, 2017.

**Supplementary Information:**

The online version contains supplementary material available at 10.1186/s13063-022-06436-0.

## Background

Since the introduction of randomized clinical trials (RCT) into clinical research, the placebo effect, as the symptom improvement following inert treatments, has become a fixed component of RCTs designed to disentangle the specific effects of a substance or treatment from the unspecific effects of the application of an inert substance [1]. When compared to the effect of drugs in randomized placebo-controlled trials, placebo effects can vary substantially, ranging from under 10% to over 60% [2–4]. Improving “assay sensitivity” by minimizing the placebo response and thus maximizing the difference between the pharmacological drug effect and the placebo response are a major desire when testing new compounds in RCTs [5]. It is a basic requirement that the full drug effect is not masked by an unspecific placebo effect. In contrast, once a drug is in routine clinical use on the market, it becomes beneficial to add a strong placebo effect on top of the pharmacological effect.

The placebo effect is composed of different factors such as the natural course of a disease or fluctuation of symptoms, response biases, effects of co-interventions, or statistical phenomena such as regression to the mean [6]. In addition, environmental factors like the surroundings and conditions of the therapy application and, in particular, the patient expectation regarding the treatment benefit are affecting the placebo response which is not limited to placebo preparations (inert substances) but also modifies the pharmacological effects of a drug [7, 8].

As pain has been shown to have a significant response to placebo treatment [6] the development of new and effective analgesic drugs depends on an optimized assay sensitivity in the clinical trial setting to demonstrate the true analgesic potential of the investigated substance. On the other hand, maximizing the placebo effect via modification of the doctor-patient communication would increase the analgesic effect in the routine clinical setting and thus help the patient [9].

Therefore, in this prospective, randomized, double-blind, placebo-controlled study, we will study in a 3-group, 2-sequence 2-period cross-over design the characteristics and extent of placebo responses triggered by a study team trained to maximize or to minimize placebo effects. The study participants will not be informed about the actual study goal and the nature of the different training stages of the study team. The study will be conducted on healthy volunteers within the highly standardized setting of analgesic therapy in a clinical trial unit as it can be found in early clinical development. Comparing the responses to verum and placebo between these three conditions will allow to answer the question whether and to what extent a trained study team can differentially influence placebo responses.

## Methods/design

**Fig. 1:**
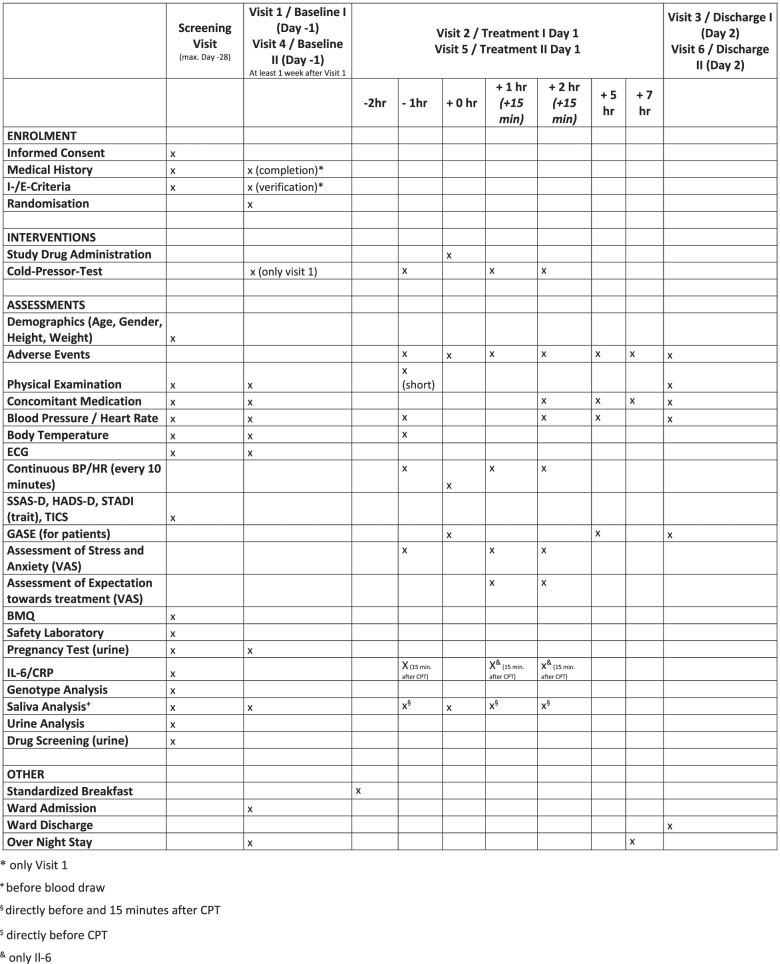
Schedule of enrolment, interventions, and assessments according to SPIRIT figure. *Only visit 1. ^**+**^Before blood draw. ^§^Directly before and 15 min after CPT. ^$^Directly before CPT. ^&^Only Il-6

The PINgPOng study is a prospective, randomized, double-blind, single-center, placebo-controlled three-arm study with a 3-group, 2-sequence, 2-period cross-over design within each study arm. A schedule of enrollment, intervention, and assessment is shown in a flowchart according to the SPIRIT 2013 Statement (Fig. 1), and the minimum content of a clinical trial protocol for an interventional trial (SPIRIT checklist) is reflected in a supplemental file. The study is conducted in the Phase I-Unit of the Institute of Clinical Chemistry and Clinical Pharmacology of the University Hospital Bonn according to the principles of ICH-GCP and the Declaration of Helsinki.

The first part of the study (arm A) will be performed with a normal acting “untrained study team,” which is blinded to the actual rationale of the study. Before the second part of the study (arm B), the study team will be informed about the true nature of the study. The study team will be trained in an “educational short course” how to increase the placebo effect by learning about the basic mechanisms initiating a placebo response (subject expectations, situational factors, verbal and non-verbal communication with the subject), how to emphasize the positive effects of the drug, and how to react to certain behaviors of the subjects. Before the third part of the study (arm C), the study team will get another “educational short course,” however, this time focusing on verbal and non-verbal communication patterns intending to decrease the placebo response. Of note, the study participants will not be informed about the actual study goal and the nature of the different training stages of the study team. Instead, they will be told that the general effects of pain perception will be analyzed. Comparing the effects between these three study parts, enrolling 32 healthy volunteers each, subjected to an experimental pain model, will allow to answer the question whether and to what extent a trained study team (neutral vs. maximize vs. minimize placebo responses) will differentially affect placebo responses.

Interindividual and group-specific differences in the analgesic effect will be assessed using the cold pressor test (CPT), which is an established and widely used experimental pain model of acute tonic pain and pain tolerance and which is sensitive to opioid analgesia [10, 11]. The study medication will be oxycodone, a semisynthetic opioid with analgesic effects on experimental pain including both acute and chronic pain conditions at a single dose of 15 mg. It has been shown to be valid in several studies on pain stimulation having a sufficient efficacy in the pain model used in this study and shown to be suitable for placebo-controlled studies [12, 13]. Typical adverse effects of opioids which might influence the expectation of the study participants to be under verum treatment need to be considered when interpreting the study results.

### Public/patient involvement

There was no patient or public involvement in the design of the protocol.

### Primary objective

The primary objective of this study is the comparison of the treatment effect, measured as the intra-individual difference of pain intensity in the cold pressor test after administration of an analgesic medication (oxycodone) or placebo assessed by the difference of the areas under the curves (AUC) of the visual analog scale (VAS) before and after the administration of the study drug with an untrained study team (arm A) and after training the study staff to optimize (arm B) or to attenuate (arm C) analgesic therapy.

### Secondary objectives

The secondary objectives of this study are the comparison of adverse effects of the study participants between the study arms, the analysis of the predictability of effects by selected genotypes (catechol-*O*-methyltransferase (COMT Val158Met), μ-opioid receptor, oxytocin receptor) and neuroendocrine factors (salivary cortisol and alpha-amylase activity (sAA)), serum level of the C-reactive protein (CRP) and interleukin-6 (IL-6), the analysis of the predictability of effects by characteristic traits (anxiety, depression, stress assessed by State-Trait-Angst-Depressions-Inventar (STADI), Hospital Anxiety and Depression Scale – German Version (HADS-D), Generic Assessment of Side Effects (GASE), Somatosensoric Amplification Scale (SSAS-D), Trierer Inventar zum chronischen Stress (TICS)), and the analysis of the predictability of effects by somatic reactions (blood pressure, heart rate).

### Inclusion criteria

The following are inclusion criteria applicable for all individuals in the three parts (treatment arms) in this study: males and females aged between 18 and 60 (inclusive) years of age at the screening examination, healthy as determined by a responsible investigator based on a medical evaluation including medical history, physical examination, laboratory tests, and 12-lead ECGs. A subject with a clinical abnormality or laboratory parameter(s) outside the reference range which seems irrelevant for the study objectives may be included in consultation with the principal investigator. All individuals need to have given their informed consent in writing.

### Exclusion criteria

The main exclusion criteria include the following: subjects who are unable to understand the nature, scope, significance, and consequences of this clinical study; subjects who are not able to understand and communicate in German as native language; subjects incapable to follow study instructions, to comply with the requirements and restrictions listed in the consent form, and to attend and complete all required visits; evidence of acute or ongoing severe infection, current chronic active disease (e.g., cardiac/pulmonary/liver/kidney/inflammatory/autoimmune diseases) as assessed by the investigator; known hormonal disease; history of a relevant psychiatric disease; history of chronic pain experience; history of any other relevant disease or condition that, in the opinion of the investigator, puts the subject or the study results at unacceptable risk or may interfere with the study procedures and results or with the subject’s participation in this clinical study; relevant concomitant medication (hormones and systematic steroid therapy except contraceptive medication, psychiatric drugs, etc.); history of hypersensitivity to the study medication or intolerance of other opioid medication; BMI < 18 or > 30 kg/m^2^ (inclusive); history of abuse of medication, drugs, or alcohol; preceeding participation in another arm of the study; and AUC < 10% in cold pressor test at baseline testing.

### Intervention

The study participants will be recruited by public advertisements and internal database search. At the screening visit, each subject has to provide written informed consent to the study. During the screening, the demographic variables, medical history, concomitant medication, and physical examination will be documented. Vital signs, electrocardiogram (ECG), and laboratory tests are obtained by a study nurse and the treating physician. Psychometric tests will be performed (STADI (trait), HADS-D, SSAS-D, TICS, BMQ). Laboratory tests include complete blood count, serum chemistry, coagulation, drug screening, genotype analysis (catechol-*O*-methyltransferase (COMT Val158Met), μ-opioid receptor, oxytocin receptor), pregnancy test and urine analysis, saliva analysis of cortisol, and alpha-amylase-activity (before blood draw). Finally, the inclusion and exclusion criteria are reviewed. Screening and baseline visits are performed within 28 days.

At the baseline visit on the day before the administration of the study drug, the subjects are admitted to the study ward, vital signs and ECG as well as the occurrence of adverse events and changes of the concomitant medication are documented, and a physical examination is performed. After an additional pregnancy testing and saliva analysis, subjects are randomized into the treatment arms A, B, and C.

On the treatment day, 1 h before study drug administration, vital signs are documented and a short physical examination is performed, a blood sample for IL-6 and CRP and saliva sample for cortisol and amylase activity is taken, and the first CPT as reference is performed. Afterwards, blood pressure and heart rate are documented every 10 min for the following 3 h. Then, 15 mg oxycodone or placebo is administered followed by taking a sample for cortisol and amylase activity and assessing the side effects with the GASE questionnaire as reference. One and 2 h later, additional CPTs are performed and a blood sample for IL-6 and CRP, and saliva sample for Cortisol and amylase-activity is taken. Subjects are assessed for the occurrence of adverse events continuously over 7 h after treatment administration. On the following day, subjects are discharged following the documentation of vital signs, assessment of adverse events, and a physical examination.

All procedures starting with the admission to the study ward are repeated for the second period of the cross-over study between 7 and 21 days after the end of the first period. As the enrolled subjects are healthy volunteers, no further medical treatment is necessary after the last trial-specific procedure. The strict schedule of the study (Fig. 1) is meant to imitate and emphasize the setting of a highly standardized inpatient clinical trial.

The intervention for each subject is terminated in case of any safety concerns like intolerable adverse events, violation of in- or exclusion criteria or pregnancy, lack of compliance and relevant protocol violations by the subject, or if the subject withdraws consent for study participation for any reason. Subjects get financial compensation for their efforts of participation. Full compensation is provided after study completion. Subjects dropping out of the study prior to the first administration of the study drug will be replaced whereas subjects dropping out of the study after the first administration of the study drug will be analyzed using all available data. Unblinding of the study treatment would be possible by opening sealed unblinding envelopes in exceptional situations.

### Allocation concealment mechanism/sequence generation

Subjects are randomly assigned to one of the blinded treatment sequences of the study medication (oxycodone – placebo or placebo – oxycodone) in a 1:1 ratio by sequential allocation of blind-labeled tablet bottles by the study staff according to a predefined computer-generated randomization list. Equal numbers of men and women will be randomized in each treatment arm (A, B, and C). The randomization list was generated by the Institute of Medical Biometrics, Informatics, and Epidemiology, University of Bonn, using the method of permuted blocks separately for the three treatment arms and stratified by gender.

### Statistical analysis

The treatment effect, difference in pain intensity under verum, and placebo treatment, will be compared between the three study arms with a two-step test procedure, testing the equality of the treatment effects in all three arms with an ANOVA first, followed by pairwise comparisons between the arms with two-sided two-sample *t*-test, in the case the primary equality hypothesis has been rejected. All tests are performed at an alpha level of 5% resulting in a family-wise error rate of 5%. The sample size is based on the expectation of the pre/post-difference in the VAS averages (AUC) to be approximately normally distributed with a standard deviation of 13 score points. Under those assumptions for a two-sided *t*-test at a level of 5%, 28 subjects per group are necessary to detect a difference between the pre/post-changes of 10% in at least two study groups with a power of 80%. To account for the loss of power due to the previous ANOVA step 32 subjects per study group will be included.

The primary target variable of the study is the change of the pain intensity assessed by the area under the curve (AUC) of the visual analog scale (VAS), measured as the percentage of the maximum possible AUC value, before and after blinded administration of 15 mg oxycodone (verum) or placebo.

The secondary target variables are the change of the time of tolerance during the cold pressor test before and after blinded administration of 15 mg oxycodone (verum) or placebo and the frequency, intensity, and quality of reported adverse events; characteristic traits (anxiety, depression, stress), selected genotypes, neuroendocrine factors, and somatic reactions will be analyzed using mixed linear models to explore the influence of various covariates on the pain perception under the different study conditions.

This clinical study will be analyzed according to the intention-to-treat (ITT) principle including all subjects that were attributed to one of the study arms and randomized to a treatment sequence. In the unlikely event of missing outcome documentation, the data will be excluded from the final analysis. All data analyzed including adverse events is documented on appropriate source data sheets and processed via electronic data capture (EDC) in a pseudonymized way. Before any data entry is performed, the study database will be validated. An audit trail will be created to provide an electronic record of which data were entered or subsequently changed, by whom, and when. The SAS software will be used to review the data for completeness, consistency, and plausibility.

## Discussion

The placebo effect—the symptom improvement following inert treatments in clinical trials—has often been considered a nuisance in clinical research. Recently, research has focused on the mechanisms underlying symptom reduction and clinical improvement observed in almost all RCTs following placebo treatments. The knowledge regarding the mechanisms orchestrating the placebo response in different physiological systems and diseases can be exploited in order to minimize the placebo response or to optimize drug-placebo differences to improve the “assay sensitivity” of clinical trials [5] as a major requirement when testing new compounds during drug development.

Patients’ expectations about treatment benefits are key modulators of health outcomes and a central mechanism of placebo responses. An individual’s expectation can both substantially shape symptoms and disease progression and influence the efficacy and tolerability of treatments [5]. The pivotal role of these processes is best illustrated by randomized-controlled clinical trials involving (inactive) placebo treatment groups. In placebo groups, changes in health outcomes cannot be explained by specific pharmacodynamic properties of a drug, rather, they are substantially determined by patients’ expectations regarding the drug treatment. Patient expectations also substantially modulate the efficacy and tolerability of active medical treatments including pharmacotherapy. Positive treatment expectation has been shown to substantially enhance the analgesic benefit from the opioid remifentanil [7]. Similar effects have been reported for other analgesic or anxiolytic treatments as well as psychotropic drugs [8].

Patients’ expectation can be influenced in different ways, in particular, through verbal instructions, the quality and quantity of verbal and non-verbal physician-patient communication, personal prior treatment experiences but also by characteristics of the therapeutic context or the intervention itself [14, 15]. Verbal information regarding the expected effects of treatments is omnipresent in the sequences of RCTs in particular. Both direct verbal and non-verbal communications with health care professionals have been shown to shape patients’ treatment expectations and treatment outcomes. This affects treatment efficacy [5, 16] and tolerability [17, 18]. For example, the pain intensity in patients with migraine did not differ in patients who received the active drug (Maxalt) but who were told the pill they received were placebos compared to those patients who received placebos but were told the pills contain the active drug [16]. In addition, in patients with irritable bowel syndrome placebo-acupuncture intervention significantly reduced symptoms but only if a warm and empathic communication with the doctor induced positive treatment expectations [19].

Placebo responses in RCTs are mainly mediated via the information subjects receive about the substance at test as well as by the verbal and non-verbal communication between subjects and the study team. For instance, it has been shown that opioid trials and a high number of planned face-to-face visits predicted the magnitude of the placebo response [20].

Therefore, in this prospective, randomized, double-blind, placebo-controlled study in a 3-group, 2-sequence, 2-period cross-over design, we will analyze the characteristics of placebo responses by systematically varying the information grade of the study team regarding the mechanisms mediating the placebo responses as well as the verbal and non-verbal communications between the study team and the subject. Comparing the responses to verum and placebo in these three study parts will allow to answer the question whether and to what extent a trained study team (neutral vs. maximize vs. minimize placebo responses) will differentially affect placebo responses and whether it would be useful to systematically utilize this approach in clinical trials.

Information on whether a subject is likely to respond to the effects of positive or negative expectations would have immediate implications for RCTs at least in the setting of an early clinical trial in healthy volunteers. However, the generalizability to the treatment of patients in clinical trials or routine clinical care may be limited. In RCTs, substantial effect sizes of placebo treatments are associated with psychological traits and state factors or genetic predisposition. For instance, it has been shown that the placebo response is associated with depressive state [21], anxiety [22, 23], and a higher level of a subject’s stress [24]. Moreover, there is some evidence that placebo effects correlate with variations in the homeostasis of opioids [25–27] and catecholamines [28]. Knowledge about the psychological trait and state factors or genetic predisposition that modulate, or even predict an individual’s response to placebo, is therefore crucial to increase assay sensitivity in RCTs and to optimally adapt therapeutic decisions in a personalized manner. So far, interindividual differences have only been investigated in studies with relatively small sample sizes, which might explain the often conflicting results [5]. We therefore also want to analyze in this study how and to what extent these factors interact with subject expectations and the placebo response created by the different approaches to handling of and communicating with the study subjects.

### Trial status

#### Recruiting

Date of recruitment start: January 15, 2018

Estimated date of recruitment completion: March 2021

Protocol version/date: April/May 22, 2019

## Supplementary Information

**Additional file 1.** SPIRIT checklist.

## Data Availability

The final trial dataset will be available for analysis for RF, MC, CC, UB, and MS. The rights to the data and for the interpretation and publication of the data remain solely with the University Hospital.

## Abbreviations

AUC: Area under the curves
BMI: Body mass index
COMT: Catechol-*O*-methyltransferase
CPT: Cold pressor test
CRP: C-reactive protein
ECG: Electrocardiogram
EDC: Electronic data capture
GASE: Generic Assessment of Side Effects
HADS-D: Hospital Anxiety and Depression Scale – German Version
ICH-GCP: International Council on Harmonisation – Good Clinical Practice
Il-6: Interleukin-6
RCT: Randomized clinical trial
SSAS-D: Somatosensoric Amplification Scale
STADI: State-Trait-Angst-Depressions-Inventar
SZB: Study Center Bonn
TICS: Trierer Inventar zum chronischen Stress
VAS: Visual analog scale
